# HIV, TB, Malaria, Filaria and Kala azar

**Published:** 2008-10

**389**

**To study the role of antioxidants in anti tubercular therapy induced hepatotoxicity and oxidative stress in patients suffereing from tuberculosis**

Agarwal M, Tekur U, Mishra TK, Khanna A, Gupta V

Maulana Azad Medical College, New Delhi, India

Hepatotoxicity associated with oxidative stress is one of the most important adverse effects of (ATT). The current management of this hepatotoxicity is to discontinue the offending drugs till the hepatic functions return to normal. The aim of present study was to assess the role of anti oxidants in alleviating this oxidative stress in tuberculosis patients.

**Materials and Methods:**

40 patients included in the study were divided into 5 groups of 8 patients each. The study period was 3 weeks. Group A consisted of normal healthy volunteers. Group B were newly diagnosed patients of pulmonary tuberculosis. Group C constituted patients with diagnosis of ATT induced hepatotoxicity (Controls). Group D were patients with ATT induced hepatotoxicity and who were prescribed Vitamin E (600mg/d) till resolution of the hepatotoxicity. Group E were patients with ATT induced hepatotoxicity and who were prescribed Vitamin C (500mg/d) in addition to Vitamin E (600mg/d) till the resolution of hepatotoxicity. Liver function tests (LFT) were done weekly. Markers of oxidative stress were measured at the beginning and end of follow up period of 3 weeks.

**Results:**

The value of malondialdehyde (MDA) decreased by 18.9% (*P*<0.05) after one month indicating that oxidative stress reduces with the improvement of disease. In the control group complete resolution of hepatotoxicity was observed on stopping ATT with decreased MDA by 24.2% at the end of 3 weeks. There was no significant difference in the MDA level between the hepatotoxic control group and those that were administered Vitamin E However, changes in GSH and MDA were 53.6% and 38.9% respectively in patients administered both Vitamin E and Vitamin C (*P*<0.05) as compared to 31% and 27% in the patients administered only Vitamin E.

**Conclusion:**

the present study shows that in ATT induced hepatotoxic patients Vitamin E and C administered in combination cause greater reduction in oxidative stress than Vitamin E administration

**390**

**Study of adverse drug reactions in various regimens under RNTCP - DOTS strategy - preliminary results**

Rao MK, Gupta PP, Chaudhry HN, Shiv Prakash, Pandey ON

BRD Medical college, Gorakhpur, (U.P.)-273013 India.

**Introduction:**

The government of India in collaboration with WHO and World- Bank launched Revised National Tuberculosis Control Programme (RNTCP) to ensure complete treatment and control of Tuberculosis as well as to reduce its social burden. The objective of attaining goals of RNTCP through DOTS therapy will not be fulfilled if its shortcomings are not evaluated and rectified within proper time limit. Keeping this in mind the present study was planned to know the adverse drug reactions(ADR) of different regimens under RNTCP - DOTS therapy at DOTS cum Microscopic Center(DMC), Nehru Hospital Of BRD Medical College, Gorakhpur.

**Aims and Objectives:**

To assess the adverse drug reactions of various RNTCP regimens under DOTS strategy in north part of eastern Uttar Pradesh.

**Methods:**

In this cross sectional study, all registered patients who were taking the treatment of tuberculosis as per criteria, guidelines and various regimens of RNTCP under DOTS strategy were observed for various ADRs and relevant findings were recorded during each clinical visit of the patients and their responses were documented. Findings were analyzed statistically.

**Results:**

In the initial 71 patients who were included in the present study, 54 were males and 17 females which shows male and female ratio of 3.2: 1. These patients were between the ages of 5-80 years. The mean age of the male patients was 32.60 ±13.84 and that of females was 24.85 ±10.58. Adverse drug reactions were found more in category 1st treatment regimens followed by category 2nd and 3rd. The mean age of the patients who had ADRs was 32±15.07. The patients who had maximum ADRs belonged to age group of 20-35 years. Mean age of males developing ADR was 34.7 ±13.49 and of females 23.75±9.92.The difference in the age of male and females who had ADRs was found to be statistically significant(df 41 =2.0545, t value <0.05).

**Conclusion:**

Our preliminary results show that Cat. 1st regimen treated patients had maximum ADRs who belonged to 20-35 age group. Maximum patients showed ADRs in first and second month of therapy. Difference in the mean age of appearance of ADRs in males and females was found to be statistically significant.

**391**

**Synthesis and antimicrobial activity of benzimidazole derivatives**

Singla Rajeev, Reddy Vijaya Bhaskara, Bhat Varadaraj G

Manipal College of Pharmaceutical sciences, Manipal University, Manipal (Karnataka), India.

**Introduction:**

Recent observations of literature suggests that the substituted benzimidazoles and related heterocyclic, which are the structural isosters of nucleotides interact easily with the polymerase and so possess potential bioactivity with lower toxicity profiles. In view of this we have undertaken synthesis and antibacterial, antifungal and antituberclar evaluation of 2-substituted phenoxymethyl benzimidazoles and 1-alkyl- 2-substituted phenoxymethyl benzimidazoles.

**Method:**

The Structures of the synthesized compounds were established with spectral characterizations using UV, IR-, ^1^H-NMR and massspectra. The Synthesized molecules were then tested against bacterial strains (E. Coli, Pseudomonas aeruginosa and coagualse positive Staphylococcus aureus), fungal strains (Candida albicans and Apergillus niger) and Mycobacterium tuberculosis in three different concentrations (1 mcg/ml, 10 mcg/ml, 100 mcg/ml). The agar diffusion method was followed to determine the activity.

**Results:**

The results of preliminary screening showed that all synthesized 2-(substituted phenoxymethyl) 1-*H* benzimidazoles and 1-alkyl-2-(substituted phenoxymethyl) benzimidazoles has potential antifungal activity against *Candida Albicans*, but only few compounds showing activity against *Apergillus niger*. Similarly all molecules showed good antibacterial activity against *E.coli* and *Pseudomonas aeruginosa*, but very few were resistance to *Staphylococcus aureus*. And all the synthesized compounds showed good activity against *Mycobacterium tuberculosis*.

**Conclusion:**

2-substituted phenoxymethyl benzimidazoles and 1-alkyl-2-substituted phenoxymethyl benzimidazoles can a potential candidates for antituberclar activity and antifungal activity.

**392**

**Comparison of cost of three different artemisin -based –combination therapies (ACT) currently used in India for acute uncomplicated multi- drugresistant falciparum malaria**

Jain C, Bansal A, Bhargava M

S.M.S. Medical College, Jaipur, Rajasthan, India.

**Introduction:**

Indiscriminate and irrational use of antimalarials has made the prevalence of resistant strains rampant across the world. Like tuberculosis, leprosy, combination therapy of malaria has also been introduced. Combination therapy of malaria is two or more schizonticidal drugs with independent modes of action and thus unrelated biochemical targets in parasite. Combination Therapy can be both artemisin- based (ACT) and non-artemisin based. This study was contemplated to compare the cost of full- course- treatment of uncomplicated MDR falciparum malaria by different ACTs. Its importance lies in treating the disease skillfully specially in countries with limited resources.

**Materials and Methods:**

Three Artemisin based combination therapies that were compared are Combination A (AS/MQ) – Artesunate / Mefloquine Combination B (AL) – Artemether – lumefantine(FDC) Combination C (AS/S/P) – Artesunate sulfadoxine + pyremethamine All drugs are expected to be given in standard doses for appropriate duration while calculating the costs.

**Results:**

It was observed that combination A costs Rs. 425-480 (depending on Pharmaceutical companies). Combination B is available as fixed dose combination and costs Rs. 324-340 for the full course. Combination C is the cheapest requiring Rs. 191-210 only for the full course.

**Conclusion:**

Amongst different ACTs for uncomplicated MDR falciparum malaria AS/ MQ combination requires 123% and A/L 70% more money to be spent in comparison to AS/S/P combination which is most economical. However cost is only one aspect of treatment. Further studies to establish relative efficacy and safety are needed before preferring one over another.

**393**

**Antispasmodic and anthelmintic activity of arbortristoside-A**

Das S, Basu S P^1^, Sasmal D^2^

^1^Noida Institute of Engineering and Technology, Greater Noida, India; ^2^Birla Institute of Technology and Sciences, Mesra, Ranchi, India.

**Objective:**

Nyctanthes arbortristis Linn is a well documented plant. Antispasmodic activity of the leaves has been established. In the present study arbortristoside-A has been isolated from the ethanolic extract of the seeds of Nyctanthes arbortristis L.

**Materials and Methods:**

The structure of the isolated compound was determined by chemical reactions and spectroscopic methods. ‘Up and down’ or ‘staircase’ method was followed for the estimation of acute toxicity of the isolated compound. The antispasmodic activity was estimated using guinea pig ileum preparation against acetylcholine, used as the spasmodic agent. The anthelmintic activity was observed according to the method described by Kailashraj and Kurup (1962).

**Results:**

From the spectral analysis the isolated compound was identified as arbortristoside-A having LD50 value, 500 mg/kg. Arbortristoside-A was found to inhibit the contractile response of acetylcholine, which shows its antispasmodic activity. Arbortristoside-A has more potent anthelmintic activity than piperazine citrate when given with atropine, but alone shows lesser activity. It is well known that the motility in worms is due to the contractile action of acetyl choline.

**Conclusion:**

The potentiation of anthelmintic activity of arbortristoside-A by atropine and inhibition of contractile action of acetyl choline indicates the anthelmintic activity of arbortristoside-A.

**394**

**Cost-effectiveness analysis of artesunate plus doxycyline versus quinine plus doxycycline in the treatment of uncomplicated *Plasmodium falciparum* malaria**

Dalvi KS, Ainapure SS, Deshmukh YA, Joseph DA, Moghe VV

Mahatma Gandhi Mission Medical College, Navi Mumbai, India.

**Introduction:**

Emergence of drug resistance to *P.falciparum* malaria becomes a leading cause of morbidity and mortality and is better treated with combination of two antimalarial drugs (as per WHO). Artesunate has certain therapeutic advantages over other antimalarial monotherapies, but the high cost of artesunate is a disadvantage in poor economic class. This study evaluates the costeffectiveness analysis of artesunate plus doxycyline (AST-DX) versus quinine plus doxycyline (Q-DX) in the treatment of uncomplicated falciparum malaria.

**Method:**

Patients of either sex (18-65 years) with evidence of uncomplicated falciparum malaria were treated randomly either with Q-DX or AST-DX and followed up on day 1, 2, 3, 7, 14, 21 and 28. Clinical and parasitological cure on day 28 was taken as effective outcome. Direct and indirect costs of each therapy were taken into account to calculate average cost-effectiveness ratio (ACER).The treatment lower ACER was considered as a costeffective treatment.

**Results:**

The result of the study show that Q-DX has a cure rate of 76% but the associated side effects raise the ancillary cost of therapy. AST-DX has high cure rate of almost 100% and there are no adverse effects associated with it. ACER of AST-DX is Rs.661 only as against that of Q-DX which is Rs.777 in which exclude the cost in those patients not cured by Q-DX. Incremental cost-effective ratio is Rs.305 for AST-DX per additional expected cases cured.

**Conclusion:**

AST-DX regimen should be recommended for the treatment of uncomplicated falciparum malaria in the drug resistance areas because of high curative effect and high cost-effectiveness.

**395**

**Evaluation of efficacy of different treatment regimens for *Plasmodium falciparum* malaria in a tertiary care hospital**

Chogtu B, Bairy KL, Adiga S, Prabhu MM

Kasturba Medical College, Manipal, India

**Introduction:**

Malaria treatment and control has been undermined by emergence and spread of drug resistant malaria worldwide thereby increasing morbidity and mortality. WHO strategies to reduce malaria related mortality by early diagnosis and effective treatment. Different regimens are being practiced in our hospital like quinine and doxycycline, artesunate and doxycycline, artesunate and mefloquine etc. Each one of the regimen has its own merits and demerits. There is scarcity of data comparing the efficacy of different regimens.

**Method:**

This was a retrospective study and involved data collection of Plasmodium falciparum malaria patients diagnosed in the year 2006 to 2008 from Medical Records department of Kasturba Medical College. The efficacy of different regimens in terms of parasite clearance, defervescence and duration of hospital stay was compared as per the case sheets of the patients.

**Results:**

The most commonly prescribed combination was artesunate and doxycycline (33%), followed by quinine and doxycycline(14%), artesunate and quinine(12%), artesunate and mefloquine(12%) and others. The efficacy of these four regimens was similar. There was no statistically significant difference between the four regimens regarding hospital stay (*P*=0.15), days for parasite clearance (*P*=0.15) and defervescence (*P*= 0.21).

**Conclusion:**

All the four commonly used regimens have similar efficacy in this study. So cost effectiveness and adverse effect profile become important criteria for the selection of a regimen. Of all these regimens quinine and doxycycline is the most cost effective and can be the first choice drug for patients who do not have any contraindication or a comorbid illness.

